# Managing sickness absence of patients with musculoskeletal pain – a cross-sectional survey of Scandinavian chiropractors

**DOI:** 10.1186/s12998-018-0230-y

**Published:** 2019-01-11

**Authors:** Mette Jensen Stochkendahl, Casper Glissmann Nim, Eleanor Boyle, Ole Kristoffer Larsen, Iben Axén, Ole Christian Kvammen, Corrie Myburgh

**Affiliations:** 1 Nordic Institute of Chiropractic and Clinical Biomechanics, Campusvej 55, DK-5230 Odense M, Denmark; 2 Department of Sports Science and Clinical Biomechanics, University of Southern Denmark, Campusvej 55, DK-5230 Odense M, Denmark; 3 Private practice, Trimveien 41B, N-3188 Horten, Norway; 4 Unit of Intervention and Implementation Research for Worker Health, Institute of Environmental Medicine, Karolinska Institutet, Nobels väg 13, 171 77 Stockholm, Sweden; 5 Private practice, Aagards plass 5, 3211 Sandefjord, Norway; 6 Institute of Health and Society, University of Oslo, Kirkeveien 166, Frederik Holsts hus, 0450 Oslo, Norway

**Keywords:** Occupational health services, Chiropractic, Return to work, Absenteeism, Policy, Work disability prevention

## Abstract

**Background:**

Musculoskeletal pain is a major cause of work disability. Many patients with musculoskeletal pain seek care from health care providers other than their general practitioners, including a range of musculoskeletal practitioners. Therefore, these musculoskeletal practitioners may play a key role by engaging in sickness absence management and work disability prevention. This study aimed to determine the prevalence of musculoskeletal practitioners’ practice behaviours, and their perceptions and beliefs about sickness absence management by using Scandinavian chiropractors as an example, as well as to examine the association between these characteristics and two different practice behaviours.

**Methods:**

As part of a mixed-methods study, we surveyed members of the national chiropractic associations in Denmark, Norway, and Sweden in 2016. Descriptive statistics were used to describe prevalence. Multilevel logistic regression with backwards stepping was used to estimate odds ratios with 95% confidence intervals between each of the two practice behaviours and the characteristics.

**Results:**

Out of the 802 respondents (response rate 56%), 372 were Danish, 349 Norwegian, and 81 Swedish. In Denmark and Norway, 38.7 and 37.8% always/often considered if sick leave was appropriate for their patient compared to 21.0% in Sweden (*p* = 0.007); and 86.5% of the Norwegian chiropractors always/often recommended to return-to-work versus 64.5 and 66.7% in Denmark and Sweden respectively (*p* < 0.001). In the final models, factors associated with the two practice behaviours were age, level of clinical experience, working as a teacher, the tendency to be updated on current legislations and policies using social services, contact with general practitioners, relevance of engagement in SAM, consideration of workplace factors, SAM as part of the clinical tool box, patient out-of-pocket fee, and recommending fast return-to-work.

**Conclusions:**

Whilst not always engaged in sickness absence management with regards to musculoskeletal pain, chiropractors favour a ‘return-to-work’ rather than a ‘stay-at-home’ approach. Several practice behaviours and perceptions and beliefs are associated with these outcomes; however, system or organisational barriers are linked to clinician non-engagement.

**Electronic supplementary material:**

The online version of this article (10.1186/s12998-018-0230-y) contains supplementary material, which is available to authorized users.

## Background

Musculoskeletal pain is a major cause of work disability with socioeconomic consequences. Back pain-related disorders alone are enormously costly and are responsible for up to one quarter of days off from work. Across Scandinavia [1, 2], four out of ten sickness certifications are based on a musculoskeletal diagnosis [3].

In many parts of Europe, health reforms have focussed on shifting secondary care services into the community. However, the effort to reduce key cost drivers, such as second level diagnostic procedures and medical specialists, has resulted in a growing pressure on the general practitioners (GP) [4, 5]. The GPs’ traditional gatekeeper role has become a particular point of stress, as this function is becoming administratively complicated and time consuming. In the area of work-related musculoskeletal healthcare, one response to this status quo has been to decentralise and delegate some functions to appropriately qualified auxiliary healthcare practitioners. Dealing with the ever-increasing demands for providing sickness certification is a contemporary example of where this kind of shift is occurring. Physiotherapists, chiropractors, and manual therapists are increasingly becoming the first point of contact and the principal provider of healthcare for individuals with musculoskeletal conditions [6, 7]. For example, private sector musculoskeletal practitioners cater to approximately 25% of the healthcare seekers for back pain in the UK [8], and at least one third of back pain patients in Denmark choose to see a chiropractor [7]. Many of these patients may not see another health practitioner about their back pain [9]. With the substantial cost implications of work disabilities for national economies and the increasing care provided exclusively by musculoskeletal practitioners, there is a large potential for integration of work disability prevention in the model of care provided by these practitioners [10]. Further, data from previous studies have indicated a potential for cost-effectiveness by including musculoskeletal practitioners in occupational health services [11–13].

This paper presents a cross-sectional, population-based survey, which is the second phase of a two-phased sequential, exploratory mixed-methods study. The first phase of the study involved a qualitative case study [14]. The interviews reported in the qualitative phase identified perceived barriers and facilitators, as well as practice behaviours of a group of musculoskeletal practitioners (chiropractors) with regards to sickness absence management (SAM) of their patients. These findings directly contributed to the development of the questionnaire used in this paper. In both phases of the study, we used a cohort comprised of Danish, Norwegian, and Swedish chiropractors as an example of a specialist healthcare practitioner group within the area of musculoskeletal health. In Denmark, Norway, and Sweden, chiropractors function as primary care sector practitioners and as first point of contact for patients with musculoskeletal disorders. The Scandinavian chiropractors receive their chiropractic education in various English-speaking countries (e.g., England, USA) or in Denmark, and are integrated at different levels in their healthcare system. But, the most noticeable difference is that since 2008, the Norwegian chiropractors have been licensed to certify sickness absence up to 12 weeks. A full overview of the differences is provided elsewhere [14].

In the first phase, we found that the chiropractors’ practice behaviour was governed by the national legislations and policies of their respective countries. The rationale for engaging in SAM was related to the perceived level of competencies, an obligation to society, and to optimise favourable patient trajectories. For some chiropractors, SAM was highly integrated in their clinical care, but for others, it was not. The perceived barriers for engaging in SAM were related to patients’ and other stakeholders’ definition of the chiropractors’ scope of practice, patient out-of-pocket expenses, the administrative burden versus level of honorarium, and the lack of communication with other stakeholders.

Building on this prior work, we sought to 1) determine the prevalence of musculoskeletal practitioners’ key practice behaviours, and perceptions and beliefs about SAM using Scandinavian chiropractors as an example, and 2) to determine what characteristics of the practitioners, practice behaviours, perceptions and beliefs, and country were associated with two different practice behaviours, i.e., how often the practitioner considers sick leave appropriate for the patient and how often the patient is recommended to return-to-work.

## Material and methods

### Study participants and procedures

We conducted a population-based, cross-sectional survey of all members of the national chiropractic organisations in Denmark, Norway, and Sweden. The national organisations cover 90–97% of all chiropractors in the three countries. Anonymised mailing lists were retrieved from the respective chiropractic organisations. By employing a series of screening questions, chiropractors were asked to complete the questionnaire if and only if, they were currently involved in patient management. Chiropractors were invited to participate via email in September 2016. A link in the email enabled the participant to directly access the survey using an online survey tool called *SurveyExact*. Two email reminders were sent to enhance the response rate (one and two weeks after the initial invitation). Further, social media outlets (i.e., Internet, Facebook and association electronic newsletters) were used to boost information about the study.

### Ethical considerations

In Denmark, the regional ethics committee of Southern Denmark gave approval for the study and declared that the study did not fall within the scope of the Medical Research Involving Human Subject Act (§14). The same conditions applied in Norway. Approval for data handling and storage covering both Denmark and Norway under the EEA-collaboration was granted from the Danish Data Protection agency. In Sweden, the regional ethics committee of Stockholm evaluated the project and found the study did not need ethical permission (advisory statement 2016/3:1).

### Designing the survey

Potential items for inclusion in the survey were informed via the interviews from phase one. Four members of the research group drafted, discussed, and selected the final questions. The survey was developed in Danish and translated into Norwegian and Swedish by three bilingual members of the research team. To assess face and content validity, a draft survey was sent to members (*n* = 5) of the researchers’ networks for comments. In order to obtain comments, a link to the pilot survey was also sent to three chiropractors with experience in SAM of musculoskeletal pain conditions (one in each of the countries). Finally, the survey was sent to the interview participants from phase one to determine whether the factors identified in the interview were satisfactorily represented in the survey.

As the result of testing the face and content validity, five questions were removed from the Swedish questionnaire because they were not applicable to the Swedish context. These questions pertained mainly to the collaboration and communication with GPs and social services, and reimbursement schemes. The social services are the public compensation agencies that provide financial compensation and return-to-work services Also, the questions relating to the sickness certification process were left in the Norwegian questionnaire only, because they are the only group that have the medico-legal rights to perform the role.

### The instrument

The final survey contained 39 questions and was divided into three sections (Additional file 1). The first question was a screening question enquiring if the respondent saw patients in the primary sector. Only if the respondent answered positively, the rest of the questionnaire unfolded. In the first section, six questions inquired about the participants’ characteristics. In the second section, there were 12 questions about practice behaviour using either Likert scales or multiple-choice items. For the 21 perceptions and beliefs questions in the third section, Likert response scales were used. In this report, variables are presented as short names and definitions are found in Tables 1, 2, and 3.

**Table 1:** Participants’ characteristics

Variable (short name in **bold**)	Total		Denmark		Norway		Sweden		*P* ^a^
	*N* = 774		*N* = 344		*N* = 349		*N* = 81		
	*n*	(%)	*n*	(%)	*n*	(%)	*n*	(%)	
**Gender**, female	339	(43.8)	185	(53.8)	122	(35.0)	32	(39.5)	< 0.001
**Age** categories, years									
21–30	130	(16.8)	37	(10.8)	90	(25.8)	3	(3.7)	< 0.001
31–40	252	(32.6)	89	(25.9)	141	(40.4)	22	(27.2)	
41–50	183	(23.6)	92	(26.7)	69	(19.8)	22	(27.2)	
51–60	140	(18.1)	90	(26.2)	27	(7.7)	23	(28.4)	
60+	65	(8.4)	34	(9.9)	20	(5.7)	11	(13.6)	
Years since graduating as a chiropractor **(Experience)**									
0–5	194	(25.1)	59	(17.2)	127	(36.4)	8	(9.9)	< 0.001
6–10	148	(19.1)	54	(15.7)	84	(24.1)	10	(12.4)	
11–15	108	(14.0)	53	(15.4)	42	(12.0)	13	(16.1)	
16–20	68	(8.8)	24	(7.0)	30	(8.6)	14	(17.3)	
21+	248	(32.4)	151	(43.9)	63	(18.1)	34	(42.0)	
Country of graduation **(graduation country)**									
Denmark	227	(32.8)	169	(49.1)	58	(16.6)	NA		< 0.001
USA	167	(24.1)	94	(27.3)	73	(20.9)	NA		
UK (excluding Wales)	242	(34.9)	68	(19.8)	174	(49.9)	NA		
Other (Australia, Canada and Wales)	50	(7.2)	9	(2.6)	41	(11.8)	NA		
In what capacity(ies), do you work as a chiropractor?^b^ **(Working as)**									
Clinic owner	497	(64.2)	220	(64.0)	214	(61.3)	63	(77.8)	0.356
Employee, private clinic	284	(36.7)	124	(36.1)	144	(41.3)	16	(19.8)	< 0.001
Intern	47	(6.1)	19	(5.5)	27	(7.7)	1	(1.2)	< 0.001
Teacher	36	(4.7)	25	(7.3)	10	(2.9)	1	(1.2)	< 0.001
Researcher	16	(2.1)	8	(2.3)	3	(0.9)	5	(6.2)	0.508
Employee, public hospital	16	(2.1)	11	(3.2)	2	(0.6)	3	(3.7)	< 0.001
Employee, insurance company	33	(4.3)	31	(9.0)	2	(0.6)	0	0	< 0.001
Other	43	(5.6)	13	(3.8)	24	(6.9)	6	(7.4)	< 0.001

^a^Chi^2^ test; ^b^Multiple answers were allowed; NA = Not applicable (i.e. the question was not asked in this country)

**Table 2:** Practice behaviours in relation to sickness absence management

Variable (short name in **bold**)	Total		Denmark		Norway		Sweden		*P* ^a^
	*N* = 774		*N* = 344		*N* = 349		*N* = 81		
	*N*	(%)	*n*	(%)	*n*	(%)	*n*	(%)	
How often do you:									
**Consider** if **sick leave** is **relevant** for your patient always/often	282	(36.4)	133	(38.7)	132	(37.8)	17	(21.0)	0.007
**Recommend** your patient **to return-to-work** rather than to stay at home always/often	578	(74.7)	222	(64.5)	302	(86.5)	54	(66.7)	< 0.001
**Consider workplace factors** when evaluating a patient always/often	711	(91.9)	315	(91.6)	321	(92.0)	75	(92.6)	0.926
**Use a fixed procedure** in the administrative part of handling sick leave, e.g. check lists, templates, written procedures always/often	78	(10.1)	8	(2.3)	62	(17.8)	8	(9.9)	< 0.001
The chiropractor **initiates a dialogue** about sick leave always/often	134	(17.3)	61	(17.7)	64	(18.3)	9	(11.1)	0.286
The chiropractor has all/most of the **competencies** needed for sick leave management	563	(72.7)	192	(55.8)	315	(90.3)	56	(69.1)	< 0.001
Which of the following stakeholders are you ***typically*** **in contact with** when handling sick leave?^b^									
General practitioners	555	(71.7)	240	(69.8)	255	(73.1)	60	(74.1)	0.321
Workplaces	288	(37.2)	85	(24.7)	166	(47.6)	37	(45.7	< 0.001
Social services	266	(38.4)	108	(31.4)	158	(45.3)	NA		< 0.001
Unions^c^	16	(2.3)	15	(4.5)	1	(0.3)	NA		0.001
Private insurance companies	104	(13.4)	65	(18.9)	23	(6.6)	16	(19.8)	< 0.001
Other stakeholders	32	(4.1)	12	(3.5)	7	(2.0)	13	(16.1)	< 0.001
Have you ever **disagreed with** any of the following stakeholders regarding sick leave of patients?^b^									
Patients	590	(76.2)	250	(72.7)	291	(83.4)	49	(60.5)	< 0.001
General practitioners	394	(50.9)	127	(36.9)	223	(63.9)	44	(54.3)	< 0.001
Workplaces	422	(54.5)	169	(49.1)	228	(65.3)	25	(30.9)	< 0.001
Social services	232	(33.5)	137	(39.8)	95	(27.2)	NA		< 0.001
Unions^c^	33	(4.8)	28	(8.1)	5	(1.4)	NA		< 0.001
Other healthcare provides	139	(17.7)	63	(18.6)	61	(17.5)	14	(17.3)	0.463
Private insurance companies	102	(13.0)	65	(18.9)	13	(3.7)	24	(29.6)	< 0.001
Other stakeholders	22	(2.8)	9	(2.6)	7	(2.0)	6	(7.4)	0.154
Do you use any of the following resources to **stay updated** with sick leave legislations? ^b^									
Post-graduate courses	178	(23.0)	27	(7.9)	144	(41.3)	7	(8.6)	< 0.001
Guidelines	416	(53.8)	123	(35.8)	257	(73.6)	36	(44.4)	< 0.001
Scientific papers	269	(34.8)	101	(29.4)	128	(36.7)	40	(49.4)	0.008
Discussions with peers	544	(70.3)	226	(65.7)	266	(76.2)	52	(64.2)	< 0.001
Health-related websites	324	(41.9)	115	(33.4)	166	(47.6)	43	(53.1)	< 0.001
Public media	315	(40.7)	157	(45.6)	102	(29.2)	56	(69.1)	< 0.001
Social media	175	(22.6)	63	(18.3)	90	(25.8)	22	(27.2)	0.009
Social services	350	(45.2)	62	(18.0)	261	(74.8)	27	(33.3)	< 0.001
Chiropractic association	429	(55.4)	168	(48.8)	224	(64.2)	37	(45.7)	< 0.001

^a^Chi^2^ test; ^b^Multiple answers were allowed; *NA* Not applicable (i.e. the question was not asked in this country); ^c^ The Scandinavian countries are highly unionized and union representatives are sometimes actively involved in the RTW process

**Table 3:** Perceptions and beliefs about sickness absence management

Variable (short name in **bold**)	Total		Denmark		Norway		Sweden		*p* ^a^
	*N* = 774		*N* = 344		*N* = 349		*N* = 81		
	*n*	(%)	*n*	(%)	*n*	(%)	*n*	(%)	
Strongly agree or Agree with the following statements:									
My patients **expect** that **I engage** in their sick leave	430	(55.6)	201	(58.4)	190	(54.4)	39	(48.2)	0.114
Usually, it is **not relevant** for me to engage in my patients’ sick leave	135	(17.4)	73	(21.2)	39	(11.2)	23	(28.4)	< 0.001
It is better to let the **general practitioner to handle the complicated cases**	316	(40.8)	131	(38.1)	154	(44.1)	31	(38.3)	0.254
It is better, if the patient is **seen by a general practitioner first**	83	(10.7)	53	(15.4)	19	(5.4)	11	(13.6)	< 0.001
Recommendations regarding sick leave is a part of my **clinical tool box**	637	(82.2)	264	(76.7)	307	(88.0)	66	(81.5)	< 0.001
**Recommending fast return-to-work** is important	681	(88.0)	293	(85.2)	316	(90.5)	72	(88.9)	0.062
It is **important to** continuously **adjust** the patient’s return-to-work needs according to their particular situation.	726	(93.8)	315	(91.6)	335	(96.0)	76	(94.0)	0.019
Managing sick leave is **burdensome**	243	(31.4)	141	(41.0)	53	(15.2)	49	(60.5)	< 0.001
Being involved in managing sick leave is **professionally satisfying**	491	(63.4)	164	(47.7)	279	(79.9)	48	(59.3)	< 0.001
The **patients’** out of pocket **expense is a barrier** for chiropractors becoming involved in sick leave management	253	(32.7)	123	(35.8)	106	(30.4)	24	(29.6)	0.185
Becoming more involved in sick leave management is **a natural development** of the chiropractic scope of practice	560	(72.4)	229	(66.6)	270	(77.4)	61	(75.3)	0.008
Having sick leave rights would increase the **professional legitimacy**	552	(71.3)	187	(54.4)	304	(87.1)	61	(75.3)	< 0.001
Chiropractors are the **best at managing** sick leave in patients with musculoskeletal complaints	551	(79.5)	238	(69.2)	313	(89.7)	NA		< 0.001
There is a **good dialogue** between the general practitioners and me	315	(45.5)	154	(44.8)	161	(46.1)	NA		0.919
I’m **adequately reimbursed** for the administrative part of managing sick leave	52	(7.5)	12	(3.5)	40	(11.5)	NA		< 0.001
How many patients do you think would you see in your practice if you took on a more **prominent role** in managing sick leave?									
Markedly more	49	(6.6)	17	(5.2)	16	(4.8)	16	(20.3)	< 0.001
A little more	235	(31.8)	65	(19.9)	131	(39.2)	39	(49.4)	
No difference	455	(61.5)	244	(74.6)	187	(56.0)	24	(30.4)	
A little fewer	0	(0)	0	(0)	0	(0)	0	(0)	
Markedly fewer	1	(0.1)	1	(0.3)	0	(0)	0	(0)	

^a^Chi^2^ test; ^b^Multiple answers were allowed; *NA* Not applicable (i.e. the question was not asked in this country)

### Data analysis

Items with Likert-scale response options were dichotomised into “Always” and “Often” versus “Sometimes,”,“Rarely,” and “Never” (practice behaviour section) or “Strongly agree” and “Agree” versus “Neither/nor,” “Disagree,” and “Strongly disagree” (perceptions and beliefs section) because of small cell sizes in some of the categories.

The prevalence of the participants’ characteristics, practice behaviours, and perceptions and beliefs were described using frequencies and compared across countries using the Chi-squared test.

Two outcomes were defined from the practice behaviour section; 1) “How often do you consider if sick leave is appropriate for your patient?” where “always” or “often” were considered a positive outcome (*consider sick leave appropriate*); and 2) “How often do you recommend your patient to return-to-work rather than to stay at home?” where “always” or “often” were considered a positive outcome (*recommend to return-to-work*).

Associations were tested in a multilevel logistic regression analysis in a three-step approach. In step 1, the association between each of the outcomes and the independent candidate variables were tested in univariate logistic regression analyses. In case of an expected frequency below 5%, categories were collapsed with the nearest category. Results were expressed using odds ratios (OR) with 95% confidence intervals (CI). Candidate variables with *p* < 0.1 were retained for step 2. In step 2, independent candidate variables identified in step 1 were tested for multicollinearity within each section*.* If multicollinearity existed, the variables with the lowest association with the outcome were excluded from the analysis. For each section, we entered all retained variables into a multilevel logistic regression and conducted backwards stepping with p to remove at 0.10. We used country as the second level. In step 3, variables that remained in the reduced section models were combined into one model for each outcome. Model fit was tested using ROC curves and calculation of area under the curve. The amount of variance explained by country was estimated using intraclass correlation. Our dataset had, in total, 2.6% missing values. No imputation was performed for these values. All statistical analyses were performed using Stata IC version 15.1 (StataCorp., Texas, USA, 2017).

## Results

The survey was issued to 1437 participants: 575 in Denmark, 653 in Norway, and 209 in Sweden. The overall response rate was 55.8% (*n* = 802). In Denmark, the response rate was 64.7% (*n* = 372), in Norway 53.4% (*n* = 349), and in Sweden 38.7% (*n* = 81). Twenty-eight respondents from Denmark did not see patients in the primary sector and were consequently excluded. The final number of respondents was therefore *n* = 774.

### Participant characteristics

Characteristics of the 774 respondents are described in Table 1. The proportion of female chiropractors was highest in Denmark (53.8% vs. 35.0% [Norway] and 39.5% [Sweden]; *p* < 0.001), and the Norwegian population was relatively the youngest (*p* < 0.001) and had graduated more recently (*p* < 0.001). More Danish chiropractors graduated in Denmark compared to Norwegian chiropractors (49.1% vs. 16.6; *p* < 0.001), whereas more Norwegian chiropractors graduated in the United Kingdom (49.9% vs. 19.8%; *p* > 0.001). In all three countries, the majority of respondents were owners of private clinics (*p* = 0.36).

### Practice behaviour

When looking at the two primary outcomes, we observed statistically significant different distributions between the three countries. In Denmark and Norway, 38.7 and 37.8% always/often considered sick leave appropriate compared to 21.0% in Sweden (*p* = 0.007); and 86.5% of the Norwegian chiropractors always/often recommended to return-to-work versus 64.5 and 66.7% in Denmark and Sweden respectively (*p* < 0.001) (Table 2). For most variables, the proportion of Norwegian chiropractors reporting SAM-supportive behaviour was higher compared to their Danish and Swedish colleagues, observed as statistically significant different distributions across the countries (Table 2). However, while more than 91% of chiropractors in all three countries always/often consider workplace factors in the evaluation of patients and more than 71% always/often have contact with the patient’s GP, less than 18% always/often initiate the dialogue about sick leave. (Table 2).

When asking the Norwegians about prescription of sick leave, 50.1% answered that they prescribe full-time sick leave once a week or more, and 38.1% always or often prescribed part-time sick leave. Only 1.7% of the Norwegian chiropractors always/often recommended full-time sick leave, but left the decision to certify the sick leave to the GP, and 33.8% considered the collaboration with GPs to be a lot better or better compared to the time before the chiropractors could issue sick leave prescriptions.

### Perceptions and beliefs

The participants’ perceptions and beliefs about SAM are described in Table 3. There were statistically different distributions of proportions in 10 out of 15 variables with a higher proportion of Norwegian participants answering in favour of SAM. The majority of chiropractors, in all three countries, strongly agreed/agreed that it is important to recommend fast return-to-work (Table 3).

In Norway, 14.6% of the chiropractors strongly agreed/agreed with the statement that the dialogue meetings with social services were adequately paid for and 47.0% strongly agreed/agreed that chiropractors should have full rights to certify sick leave (i.e., beyond 12-weeks). Forty-two percent strongly agreed/agreed that the GPs accept the sick leave certification rights of chiropractors, and 37.2% of the chiropractors agreed that GPs are helpful in sick leave reporting.

### Univariate analysis

Based on the univariate analysis, 22 variables were associated with the outcome *consider if SL was appropriate* at a *p* < 0.10 and were thus, included in the multivariable analysis. Three variables pertained to the participants’ characteristics (i.e., Age; Experience; Working as a teacher); 10 variables pertained to practice behaviour (i.e., Considers workplace factors; Initiates a dialogue; Competencies; Staying updated using guidelines or social services; Typically in contact with GPs; Disagreed with patients, GPs, or workplaces); and nine variables pertained to the participants’ perceptions and beliefs (i.e., Expect I engage; Not appropriate; Seen by a GP first; Clinical tool box; Important to adjust; Burdensome; Patients’ expense is a barrier; Natural development).

With respect to the outcome *recommend to return-to-work,* four variables pertaining to participants’ characteristics were associated with this outcome (Age; Experience; Working as a teacher; Working for an insurance company); 13 variables associated with the outcome pertained to practice behaviour (Initiates a dialogue; Staying updated using courses, guidelines, discussions with peers, public media, chiropractic associations or social services; Typically in contact with GPs or the workplaces; Disagreed with patients, GPs, workplaces or insurance company); and four variables from the perceptions and beliefs block (Seen by a GP first; Clinical tool box; Recommend fast return-to-work; Professional legitimacy). In total, 21 variables were associated with this outcome.

### Multivariable analysis

In the final model of factors associated with the outcome *consider sick leave appropriate* (Table 4), participants answering *Working as a teacher* and *Not appropriate [strongly agree]* were more likely to consider if sick leave was appropriate*,* and those answering *Experience of 21+ years; Consider workplace factors [often]; Clinical tool box [neutral or negative]; Patients’ expense is a barrier [agree or neutral]* were less likely to consider if sick leave was appropriate. The amount of variance in the final model explained by country was 9.2% (95% CI [1.5; 41.0]).

**Table 4:** Characteristics, with odds ratios (OR) and confidence intervals (CI), associated with the outcome *“How often do you consider if sick leave is relevant for your patient?”* (always/often versus sometimes/rarely/never) in 774 Scandinavian chiropractors, analysed using a multilevel logistic regression analysis

Characteristics	OR	[95% CI]	*p*
Experience, Years since graduation as a chiropractor			
0–5 years	Reference level	Reference level	
6–10 years	0.86	[0.51; 1.44]	0.003
11–15 years	0.95	[0.53; 1.69]	
16–20 years	1.02	[0.52; 2.01]	
21 years or more	0.40	[0.24; 0.67]	
Working as a teacher	2.45	[1.09; 5.52]	0.031
Consider workplace factors when evaluating a patient			
Always	Reference level	Reference level	
Often	0.61	[0.42; 0.89]	0.039
Sometimes	0.53	[0.21; 1.34]	
Rarely or never	1.82	[0.27; 12.42]	
Use social service to stay updated regarding sick leave legislation	1.19	[0.78; 1.83]	0.42
Typically in contact with general practitioner	1.21	[0.78; 1.88]	0.38
Has disagreed with the patient regarding sick leave	1.60	[0.97; 2.64]	0.67
Usually, it is not relevant to engage in my patients’ sick leave			
Strongly agree	Reference level	Reference level	
Agree	0.67	[0.19; 2.37]	< 0.001
Neither agree nor disagree	1.24	[0.37; 4.12]	
Disagree	2.91	[0.91; 9.30]	
Strongly disagree	3.87	[1.13 13.27]	
Recommendations regarding sick leave is a part of my clinical tool box			
Strongly agree	Reference level	Reference level	
Agree	0.83	[0.56; 1.24]	0.054
Neither agree nor disagree	0.44	[0.20; 0.99]	
Disagree	0.19	[0.05; 0.75]	
Strongly disagree	1.39	[0.25; 7.60]	
The patients’ out of pocket expense is a barrier for chiropractors becoming involved in sick leave management			
Strongly agree	Reference level	Reference level	
Agree	0.34	[0.17; 0.66]	< 0.001
Neither agree nor disagree	0.46	[0.25; 0.88]	
Disagree	0.53	[0.27; 1.03]	
Strongly disagree	1.70	[0.65; 4.43]	
Greater involvement is a natural development			
Strongly agree	Reference level	Reference level	
Agree	0.84	[0.54; 1.32]	0.24
Neither agree nor disagree	0.58	[0.32; 1.03]	
Disagree	0.39	[0.11; 1.39]	
Strongly disagree	1.39	[0.29; 6.65]	

In the final model of factors associated with the outcome *Recommend to return-to-work* (Table 5), participants answering *Working as a teacher; Staying updated using social service; Typically in contact with GP*) were more likely to recommend return-to-work, whereas those answering *Age of 60+ years; Recommending fast return-to-work [agree or neutral/negative]*) were less likely to recommend return-to-work. The amount of variance that was explained by country was 3.8% (95% CI [4.0; 27.0%]). The amount of variance that was explained by country was 3.8% (95% CI [4.0; 27.0%]).

**Table 5:** Characteristics, with odds ratios (OR) and confidence intervals (CI), associated with the outcome *“How often do you recommend your patient to return-to-work rather than to stay at home”* (always/often versus sometimes/rarely/never) in 774 Scandinavian chiropractors, analysed using a multilevel logistic regression analysis

Characteristic	OR	[95% CI]	
Age category, years			
21–30 years	Reference level	Reference level	
31–40 years	0.57	[0.31; 1.05]	0.017
41–50 years	1.08	[0.54; 2.14]	
51–60 years	0.67	[0.33; 1.35]	
60+	0.36	[0.16; 0.81]	
Working as a teacher	3.48	[1.01; 11.97]	0.048
Who initiates a dialogue about sick leave?			
Always patient	Reference level	Reference level	
Most often patient	1.15	[0.35; 3.72]	0.081
50–50	0.76	[0.24; 2.35]	
Most often or always chiropractor	0.52	[0.16; 1.72]	
Use peer discussions to stay updated regarding sick leave legislation	1.49	[0.96; 2.30]	0.074
Use public media to stay updated regarding sick leave legislation	0.68	[0.45; 1.01]	0.058
Use social service to stay updated regarding sick leave legislation	1.86	[1.14; 3.05]	0.013
Typically in contact with general practitioner	1.59	[1.04; 2.44]	0.031
It is important to recommend fast return-to-work			
Strongly agree	Reference level	Reference level	
Agree	0.46	[0.30; 0.69]	< 0.001
Neither/nor, disagree or strongly disagree	0.18	[0.10; 0.33]	

## Discussion

This study investigated the extent to which Scandinavian chiropractors currently engage in SAM with regards to musculoskeletal pain with their patients and evaluated their perceptions and beliefs about integration of work-related factors in their scope of SAM practice. Our data indicated that not all Scandinavian chiropractors engage in SAM, with less than 40% who would always/often consider if sick leave were appropriate for their patients, and 65–87% who always/often recommended their patients to return-to-work rather than to stay at home. However, and perhaps most interestingly, we observed that with chiropractors in Norway who have the right to prescribe sick leave, only about a third reported prescribing part-time sick leave, but they more often recommended their patients to return-to-work. In addition, the Norwegian chiropractors consistently reported more positive perceptions and beliefs towards SAM and a greater level of involvement with the process compared with the Danish and Swedish chiropractors.

Given the different legislations regarding sickness certification rights, we forced country of practice into the analysis throughout, but the amount of variance in the final variable models explained by country was small. In a multivariable model, factors from three sectors: participants’ characteristics; practice behaviour; and perceptions and beliefs, influenced whether Scandinavian chiropractors considered sick leave appropriate for their patients and whether they recommend the patient to return-to-work. In the model of the outcome, “How often do you consider if sick leave is appropriate for your patient?” the strongest associations were found for strongly disagreeing with the statements, “Usually, it is not relevant to engage in my patients’ sick leave” (positive association) and “Recommendations regarding sick leave is a part of the clinical tool box” (negative association). In the model relating to the outcome, “How often do you recommend your patient to return-to-work rather than to stay at home,” the strongest associations were found for “working as a teacher” (positive association) and answering neither/nor to the statement, “It is important to recommend fast return-to-work” (negative association).

Generally, we saw the highest proportion of SAM-supportive behaviour in Norway and the most positive perceptions and beliefs. Based on the findings in the qualitative part of this study [14], we believe this is a direct reflection of the legislative SAM rights and accompanying supportive system provided in Norway. The chiropractors in Norway are officially recognised as legitimate SAM partners. They have direct electronic access to the social services and are reimbursed for their services. Further, it is mandatory to engage in post-graduate education about SAM. It is likely that these factors act as facilitators or catalysts to the Norwegians’ involvement in SAM and staying updated, but due to the cross-sectional design of this study, this remains speculative.

One characteristic, “Working as a teacher,” was retained in both multivariable models and was positively associated with the outcomes. Having more than 21 years of clinical experience and being older than 60 years of age were negatively associated with the outcomes, meaning that chiropractors with these characteristics were less likely to engage in SAM. These may be spurious findings, or they may be reflective of a generally different perception of the chiropractic scope of practice and patient management. Many of the teachers are employed by the universities and thus strongly influenced by evidence-based practice under the bio-psycho-social model. This may also be the case for the younger or more recent graduates. However, the design of the study did not allow us to explore these issues in detail.

### Comparison with other studies

The integration of work-related factors in clinical practice has previously been investigated in Dutch and Canadian physiotherapists [15, 16]. Like the Scandinavian chiropractors, the physiotherapists, to a large extent, integrate work-related factors in their clinical SAM decision-making. However, in these settings, there also appeared to be system-related and organisational barriers such as, lack of communication with other healthcare providers, inability to upgrade their knowledge and competencies on SAM, and the lack of suitable reimbursement for services. These barriers were perceived to hamper the providers in integrating work-related factors in their clinical practice [15, 16].

In a study of Scandinavian GPs, no difference was found among Danish, Norwegian, and Swedish GPs’ decisions to grant sick leave when using case vignettes [17], and the GPs’ decisions to grant sick leave was more based on patients’ characteristics and beliefs about the patients’ health situation and perceived ability to work than the country of their practice. The influence of country on our findings is likely due to the different legislations under which Scandinavian chiropractors function, unlike the GPs’ legislation for these countries, which is the same.

In the case of back pain, which lies within the chiropractors’ main scope of practice [18, 19], evidence suggests that healthcare providers’ attitudes and beliefs influence their advice and recommendations about activity and work that they offer to their patients [20]. Healthcare practitioners’ beliefs of fear avoidance are associated with reported sick leave prescription, whereas a predominantly biomedical orientation under the biopsychosocial model of care is not associated with the number of sickness certificates [20, 21]. Although some studies have suggested that chiropractors may hold views or provide services, which bias toward a biomedical emphasis [22, 23], Innes et al. found that a sample of Australian chiropractors demonstrated similar levels of biomedical emphasis to that of GPs and physiotherapists from differing cultures and educational backgrounds [22]. Further to that, our results indicated that, at least to some degree, they include social aspects in their assessment of their patients.

Our results indicate that the respondents often, and independently of country, considered work-related factors as part of their clinical assessment, but less than 40% of the chiropractors in all three countries always/often considered whether sick leave was appropriate for their patient, and fewer than 50% of the chiropractors made contact with the employer when discussing SAM. These are consistent with results from private musculoskeletal practitioners in the United Kingdom [24] where it was found that these practitioners did not consider work-related issues as part of their scope of practice, and many did not regard establishing contact with the workplace as part of their role.

### Strengths and limitations

We surveyed the chiropractic population in three Scandinavian countries with a response rate between 39 and 65%. Due to the nature of the study, we do not have information about non-responders. However, the Danish and Norwegian samples were comparable to the respondents of national surveys of the profession regarding gender, age, and country of education. In Denmark, 77% responded to a 2014-survey [18]. In Norway, 61% responded to a 2011-survey [19]. Similar data are not available for Swedish chiropractors. Additionally, we are unable to evaluate how representative the Swedish sample was or provide information about non-responders.

The survey is limited to self-reported behaviour and to questions that imply some degree of generalisability across a variety of situations by using words such as “typically”. Self-reported behaviour may not necessarily reflect observed behaviour. Thus, the results of this study should only be interpreted as the practitioners’ self-reported practice behaviours under the given condition of a “typical” situation. The outcome measures and independent variables can be considered as proxy measures of behaviour. They may cover a range of constructs, and we were not able to assess how these items reflect objective measures of behaviour and practice.

### Clinical and research implications

This study contributes with novel insights about practice behaviour, and perceptions and beliefs in a particular group of musculoskeletal practitioners (chiropractors). We have identified potential avenues for increasing the involvement of this group of practitioners in sickness absence management, such as the resources the chiropractors use to stay updated about musculoskeletal-related SAM, but also some of the system barriers for engagement. The results hold the potential to highlight important system and organisational strategies useful for informing policy and practice. Given the similarities of findings in studies of GPs and private musculoskeletal practitioners in the United Kingdom and the Netherlands, we cautiously believe the findings in this study may be generalisable to other groups of healthcare practitioners in similar settings. Healthcare practitioners, researchers, and policy makers who strive to test or implement strategies in relation to work disability prevention in clinical settings should be aware that practice behaviour is not only influenced by perceptions and beliefs, but also by organisational and system factors and are linked to legislation policies and financial schemes.

## Conclusions

Whilst not always engaged in sickness absence management with regards to musculoskeletal pain, chiropractors favour a ‘return-to-work’ rather than a ‘stay-at-home’ approach. Norwegian chiropractors who have the right to prescribe sick leave consistently report more positive perceptions and beliefs towards SAM and a greater level of involvement with the process compared with the Danish and Swedish chiropractors who do not have these rights. Several practice behaviours and perceptions and beliefs are associated with these outcomes; however, system or organisational barriers are linked to clinician non-engagement.

## Additional file

Additional file 1: Survey of Scandinavian chiropractors, English version. (PDF 119 kb)

## Abbreviations

CI: Confidence interval.
GP: General practitioner.
OR: Odds ratio.
SAM: Sickness absence management.

## References

[1] Ekman M, Johnell O, Lidgren L (2005). The economic cost of low back pain in Sweden in 2001. Acta Orthop.

[2] Mortensen OS, Andersen JH, Ektor-Andersen J, Eriksen HR, Fallentin N, Frost P, et al. White paper on sickness absence and return-to-work due to musculoskeletal disorders – causes and options [in Danish]. Copenhagen: National Research Centre for the working environment; 2008.

[3] The Norwegian Labour and Welfare Administration (NAV). Statistics on certified sickness absence 2017 [in Norwegian]. https://www.nav.no/no/NAV+og+samfunn/Statistikk/Sykefravar+-+statistikk/Tabeller/legemeldte-sykefrav%C3%A6rsdagsverk-2-kv-2008-2017-diagnose-og-kj%C3%B8nn. Acceessed 22 Dec 2018.

[4] Welsh VK, Mallen CD, Wynne-Jones G, Jinks C (2012). Exploration of GPs' views and use of the fit note: a qualitative study in primary care. Br J Gen Pract.

[5] King O, Nancarrow SA, Borthwick AM, Grace S (2015). Contested professional role boundaries in health care: a systematic review of the literature. J Foot Ankle Res.

[6] Wolsko PM, Eisenberg DM, Davis RB, Kessler R, Phillips RS (2003). Patterns and perceptions of care for treatment of back and neck pain: results of a national survey. Spine (Phila Pa 1976).

[7] Lønnberg F (1997). The management of back problems among the population. I. Contact patterns and therapeutic routines [in Danish]. Ugeskr Laeger.

[8] Haetzman M, Elliott AM, Smith BH, Hannaford P, Chambers WA (2003). Chronic pain and the use of conventional and alternative therapy. Fam Pract.

[9] Pincus T, Woodcock A, Vogel S (2010). Returning back pain patients to work: how private musculoskeletal practitioners outside the national health service perceive their role (an interview study). J Occup Rehabil.

[10] Johnston V, Beales D (2016). Enhancing direct access and authority for work capacity certificates to physiotherapists. Man Ther.

[11] Phillips CJ, Phillips Nee Buck R, Main CJ, Watson PJ, Davies S, Farr A (2012). The cost effectiveness of NHS physiotherapy support for occupational health (OH) services. BMC Musculoskelet Disord.

[12] Salisbury C, Foster NE, Hopper C, Bishop A, Hollinghurst S, Coast J (2013). A pragmatic randomised controlled trial of the effectiveness and cost-effectiveness of 'PhysioDirect' telephone assessment and advice services for physiotherapy. Health Technol Assess.

[13] Desmeules F, Roy JS, MacDermid JC, Champagne F, Hinse O, Woodhouse LJ (2012). Advanced practice physiotherapy in patients with musculoskeletal disorders: a systematic review. BMC Musculoskeletl Disord.

[14] Stochkendahl MJ, Larsen OK, Nim CG, Axen I, Haraldsson J, Kvammen OC (2018). Can chiropractors contribute to work disability prevention through sickness absence management for musculoskeletal disorders? - a comparative qualitative case study in the Scandinavian context. Chiropr Man Therap.

[15] Oswald W, Hutting N, Engels JA, Bart Staal J, Nijhuis-van der Sanden MWG, Heerkens YF (2017). Work participation of patients with musculoskeletal disorders: is this addressed in physical therapy practice?. J Occup Med Toxicol.

[16] Hudon A, Laliberte M, Hunt M, Feldman DE (2015). Quality of physiotherapy services for injured workers compensated by workers' compensation in Quebec: a focus group study of physiotherapy professionals. Healthc Policy.

[17] Maeland S, Werner EL, Rosendal M, Jonsdottir IH, Magnussen LH, Lie SA (2013). Sick-leave decisions for patients with severe subjective health complaints presenting in primary care: a cross-sectional study in Norway, Sweden, and Denmark. Scand J Prim Health Care.

[18] Nielsen OL, Kongsted A, Christensen HW (2015). The chiropractic profession in Denmark 2010-2014: a descriptive report. Chiropr Man Therap.

[19] Kvammen OC, Leboeuf-Yde C (2014). The chiropractic profession in Norway 2011. Chiropr Man Therap.

[20] Darlow B, Fullen BM, Dean S, Hurley DA, Baxter GD, Dowell A (2012). The association between health care professional attitudes and beliefs and the attitudes and beliefs, clinical management, and outcomes of patients with low back pain: a systematic review. Eur J Pain.

[21] Watson PJ, Bowey J, Purcell-Jones G, Gales T (2008). General practitioner sickness absence certification for low back pain is not directly associated with beliefs about back pain. Eur J Pain.

[22] Innes SI, Werth PD, Tuchin PJ, Graham PL (2015). Attitudes and beliefs of Australian chiropractors' about managing back pain: a cross-sectional study. Chiropr Man Therap.

[23] Beliveau PJH, Wong JJ, Sutton DA, Simon NB, Bussieres AE, Mior SA (2017). The chiropractic profession: a scoping review of utilization rates, reasons for seeking care, patient profiles, and care provided. Chiropr Man Therap.

[24] Pincus T, Greenwood L, McHarg E (2011). Advising people with back pain to take time off work: a survey examining the role of private musculoskeletal practitioners in the UK. Pain.

