# Supplementary material for: Managing sickness absence of patients with musculoskeletal pain – a cross-sectional survey of Scandinavian chiropractors
Source: Chiropr Man Therap. 2019 Jan 11;27:1. doi: 10.1186/s12998-018-0230-y (PMC6329073; doi:10.1186/s12998-018-0230-y)
Supplement: Supplementary file 1 — Survey of Scandinavian chiropractors, English version. (PDF 119 kb) [file 12998_2018_230_MOESM1_ESM.pdf]

Survey of Scandinavian chiropractors, English version

Item no.	Question	Response option
**Section 1: Participants characteristics**		
1	Do you work in private practice?	(1) Yes<br>(2) No
2	Sex	(1) Female<br>(2) Male
3	Age (years)	(1) 21-30 years<br>(2) 31-40 years<br>(3) 41-50 years<br>(4) 51-60 years<br>(5) 61-70 years<br>(6) >70 years
4	Number of years since graduation as chiropractor?	(1) 0-5 years<br>(2) 6-10 years<br>(3) 11-15 years<br>(4) 16-20 years<br>(5) >21 years
5	In which capacity(ies), do you work as a chiropractor?	(1) Owner of private clinic – yes/no<br>(2) Employed in private clinic – yes/no<br>(3) Intern – yes/no<br>(4) Teacher – yes/no<br>(5) Researcher – yes/no<br>(6) Public employee – yes/no<br>(7) Employed by insurance company – yes/no<br>(8) Other– yes/no
6	In which country, did you graduate?	(1) UK<br>(2) Denmark<br>(3) USA<br>(4) Canada<br>(5) Australia<br>(6) Other

**Section 2: Practice behaviours**		
7	How often do you consider if sick leave is relevant for your patient?	(1) Always<br>(2) Often<br>(3) Sometimes<br>(4) Rarely<br>(5) Never
8	How often do you recommend your patient go to work rather than to stay at home?	(1) Always<br>(2) Often<br>(3) Sometimes<br>(4) Rarely<br>(5) Never
9	How often do you consider workplace factors when evaluating a patient?	(1) Always<br>(2) Often<br>(3) Sometimes<br>(4) Rarely<br>(5) Never
10	How often do you prescribe sick leave? (Norway only)	(1) Several times a week<br>(2) Once a week<br>(3) 1-2 times a month<br>(4) Less than once a month<br>(5) Never
11	How often do you recommend sick leave, but leave the decision up for the general practitioner?	(1) Always<br>(2) Often<br>(3) Sometimes<br>(4) Rarely<br>(5) Never

12	How often do you use a fixed procedure in the administrative part of handling sick leave, e.g. check lists, templates, written procedures?	(1) Always<br>(2) Often<br>(3) Sometimes<br>(4) Rarely<br>(5) Never
13	How often do you prescribe part-time sick leave? (Norway only)	(1) Always<br>(2) Often<br>(3) Sometimes<br>(4) Rarely<br>(5) Never
14	Who initiates a dialogue about sick leave?	(1) The patient always initiates the dialogue<br>(2) The patient often initiates the dialogue<br>(3) It is 50-50<br>(4) I often initiate the dialogue<br>(5) I always initiate the dialogue
15	How would you rate your competencies in handling sick leave?	(1) I have all the relevant competencies<br>(2) I have most of the relevant competencies<br>(3) I have half of the relevant competencies<br>(4) I have few of the relevant competencies<br>(5) I have no relevant competencies
16	Do you use any of the following resources to stay updated regarding sick leave legislation?	(1) Post graduate courses – yes/no<br>(2) Guidelines– yes/no<br>(3) Scientific papers– yes/no<br>(4) Discussions with peers – yes/no<br>(5) Social media – yes/no<br>(6) Other media (Newspapers/tv) – yes/no<br>(7) Health related websites– yes/no<br>(8) Social services– yes/no<br>(9) Chiropractic association– yes/no
17	Which of the following stakeholders are you *typically* in contact with when handling sick leave (e.g. telephone conversation, electronic communication, meetings)?	(1) General practitioner – yes/no<br>(2) Work place – yes/no<br>(3) Social service – yes/no (DK and N only)<br>(4) Union – yes/no (DK and N only)<br>(5) Insurance company – yes/no<br>(6) Other stakeholders– yes/no

18	Have you ever disagreed with any of the following regarding sick leave of patients?	(1) Patient – yes/no<br>(2) General practitioner– yes/no<br>(3) Work place– yes/no<br>(4) Social service – yes/no (DK and N only)<br>(5) Union – yes/no (DK and N only)<br>(6) Insurance company – yes/no<br>(7) Other healthcare providers – yes/no<br>(8) Other stakeholders – yes/no
**Section 3: Perceptions and beliefs**		
	**How much do you agree with the following statement?**	
19	My patients expect that I engage in their sick leave	(1) Strongly agree<br>(2) Agree<br>(3) Neither/nor<br>(4) Disagree<br>(5) Strongly disagree
20	Usually, it is not relevant to engage in my patients' sick leave	do
21	It is better to let the general practitioner handle complicated cases	do
22	It is better, if the patient is seen by a general practitioner first	do
23	Recommendations regarding sick leave is a part of the clinical tool box	do
24	Recommending fast return to work is important	do
25	It is important to continuously adjust the patient's return-to-work needs according to their particular situation.	do
26	Managing sick leave is burdensome	do

27	Being involved in managing sick leave is professionally satisfying	do
28	I'm adequately reimbursed for the administrative part of managing sick leave (DK and N only)	do
29	I'm adequately reimbursed for participating in dialogue meetings (N only)	do
30	The patients' out of pocket expense is a barrier for chiropractors becoming involved in sick leave management	do
31	Becoming more involved in sick leave management is a natural development of the chiropractic scope of practice	do
32	Sick leave rights would increase the professional legitimacy	do
33	Chiropractors are the best at managing sick leave in patients with musculoskeletal complaints (N and DK only)	do
34	Chiropractors should be granted full sick leave rights (> 12 weeks)	do
35	The general practitioner should be responsible for managing sick leave (N only)	do
36	The GP supports my work in managing sick leave (N only)	do
37	There is a good dialogue between the general practitioner and I (N and DK only)	do
38	How many patients do you think would see you in your practice if you took on a more prominent role in managing sick leave?	(1) Markedly more<br>(2) A little more<br>(3) No difference<br>(4) A little fewer<br>(5) Markedly fewer
39	Following the rights to sick list patients, how do you perceive the collaboration between chiropractors and GPs? (N only)	(1) Much better<br>(2) A little better<br>(3) No difference<br>(4) A little worse<br>(5) Much worse<br>(6) Don't know
